# Bring your own camera to the trap: An inexpensive, versatile, and portable triggering system tested on wild hummingbirds

**DOI:** 10.1002/ece3.3040

**Published:** 2017-05-18

**Authors:** Alejandro Rico‐Guevara, James Mickley

**Affiliations:** ^1^ Department of Integrative BiologyUniversity of CaliforniaBerkeleyCAUSA; ^2^ Department of Ecology and Evolutionary BiologyUniversity of ConnecticutStorrsCTUSA

**Keywords:** camera trap, field equipment, high‐speed video, hummingbirds, remote cameras, video monitoring, video triggers

## Abstract

The study of animals in the wild offers opportunities to collect relevant information on their natural behavior and abilities to perform ecologically relevant tasks. However, it also poses challenges such as accounting for observer effects, human sensory limitations, and the time intensiveness of this type of research. To meet these challenges, field biologists have deployed camera traps to remotely record animal behavior in the wild. Despite their ubiquity in research, many commercial camera traps have limitations, and the species and behavior of interest may present unique challenges. For example, no camera traps support high‐speed video recording. We present a new and inexpensive camera trap system that increases versatility by separating the camera from the triggering mechanism. Our system design can pair with virtually any camera and allows for independent positioning of a variety of sensors, all while being low‐cost, lightweight, weatherproof, and energy efficient. By using our specialized trigger and customized sensor configurations, many limitations of commercial camera traps can be overcome. We use this system to study hummingbird feeding behavior using high‐speed video cameras to capture fast movements and multiple sensors placed away from the camera to detect small body sizes. While designed for hummingbirds, our application can be extended to any system where specialized camera or sensor features are required, or commercial camera traps are cost‐prohibitive, allowing camera trap use in more research avenues and by more researchers.

## 1 INTRODUCTION

Studying animals in their natural habitat is advantageous and preferable to working with them in captivity for a variety of research questions. Natural habitats allow for the study of behavior in situ, offering opportunities to measure energetic performance of free‐living animals, and opening doors to understanding intra and interspecific interactions. Researchers face three main challenges while working with wild animals: (1) neutralizing the observer effect (Baker & McGuffin, 2007; Wade, Zalucki, & Franzmann, 2005), (2) dealing with long waiting times (review in Cutler & Swann, 1999), and (3) compensating for human sensory limitations (Weale, 1961). These challenges are solved with camera traps (reviews in Cutler & Swann, 1999; O'Connell, Nichols, & Karanth, 2011; Rowcliffe & Carbone, 2008), which are used to detect animals in a given area (Karanth, 1995; Silveira, Jácomo, & Diniz‐Filho, 2003), and to study behavior (Bischof, Ali, Kabir, Hameed, & Nawaz, 2014; Gula, Theuerkauf, Rouys, & Legault, 2010; Ohashi, D'Souza, & Thomson, 2010). Nevertheless, commercial camera traps are effective with relatively few taxa and can be prohibitively expensive (Meek & Pittet, 2012). Our goal was to design an alternative with the flexibility to study a larger variety of taxa at the lowest cost possible.

We developed a system with many advantages over available camera traps: (1) it functions mechanically and can be coupled with cameras that do not support remote triggering, (2) it can incorporate most sensors, which allows for other modes of detection besides movement (e.g., light, color, sound), (3) it offers versatility to position multiple sensors separately from the camera, adapting to the subject of interest, (4) it is inexpensive enough to be affordable in field projects requiring multiple camera traps, (5) as it is not married to any particular camera, it can be updated when a given technology becomes obsolete, (6) it is powered by standard AA batteries for long durations, facilitating recharging, easy replacement, and accessibility in remote locations, (7) it is weatherproof, light, and portable, allowing for the deployment of several units with few personnel, and (8) the triggering and sensor systems are easy to customize in the field to adapt to changing conditions or objectives.

Most camera traps have been designed to capture large animals using passive infrared motion sensors (PIR) (Meek & Pittet, 2012; Rovero, Zimmermann, Berzi, & Meek, 2013; Welbourne, Claridge, Paull, & Lambert, 2016), although some applications have been aimed at small mammals (Pearson, 1960; Soininen & Jensvoll, 2015; Villette, Krebs, Jung, & Boonstra, 2016), birds (Bolton, Butcher, Sharpe, Stevens, & Fisher, 2007; Kross & Nelson, 2011), and arthropods (review in Steen & Ski, 2014). PIR sensors detect a change in surface temperatures, such as when an animal with a different surface temperature than the background enters the scene (Welbourne et al., 2016). Despite broad use, PIR sensors present various problems such as false negatives when the subject's surface temperature differs minimally from the background (Welbourne et al., 2016), or false positives from vegetation blowing in the wind (Rovero et al., 2013; Welbourne et al., 2016). Innovations for improving detection of smaller (or poikilothermic) taxa include active infrared sensors (AIR) that trigger when an infrared beam is crossed (Hernandez, Rollins, & Cantu, 1997; Rovero et al., 2013; Swann, Hass, Dalton, & Wolf, 2004), cameras with video motion detection that trigger when there is movement in the selected field of view (Bolton et al., 2007; Kross & Nelson, 2011), and multiple sensors to improve detectability (Meek & Pittet, 2012). Unlike PIR, the former two innovations are not affected by surface temperature of the background or subject. In addition to sensor limitations, available camera traps generally lack specialized features such as tele‐macro and high‐speed video. External sensors and triggering systems (e.g., Trailmaster^®^, Cognisys^®^) provide control over the sensors used, and allow the use of some specialized cameras (Brooks, 1996; Hernandez et al., 1997; Kucera & Barrett, 1993), but all require cameras that can be remotely triggered.

A review of the market found no suitable camera trap for capturing high‐speed video of hummingbird floral visits: limitations included a combination of slow trigger speed (latency to start recording 0.5 s, cf. Meek & Pittet, 2012), low video frame rate (<60 frames/s), and no remote triggering. Our system overcomes these limitations by pairing with nearly any camera or sensor setup, allowing a researcher to choose optimal camera and sensor configurations separately to match their organism and application. We tested the system by tailoring it specifically to hummingbirds, but as it is presented here, it can be coupled to any kind of sensor and camera in order to study a wide variety of subjects (e.g., AIR sensors and night‐vision cameras to study nocturnal poikilotherms).

**Figure 1:**
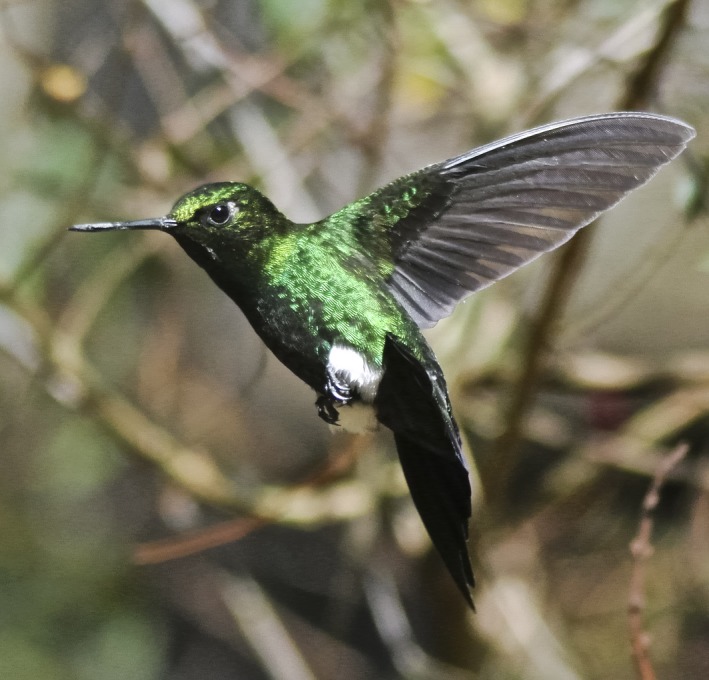
A hummingbird photographed using one of the triggering systems. A male Glowing puffleg (*Eriocnemis vestita*) captured by setting a camera in burst mode and triggered automatically by one of the systems described in the present study. A complete list of the hummingbirds studied is available in Table S3

We present a test of this novel system using hummingbirds (Figure 1). These small, superb fliers visit flowers quickly without perching–difficult subjects for camera traps to detect and record. We have studied hummingbirds drinking nectar from artificial feeders (Rico‐Guevara & Rubega, 2011) and have developed predictions from biomechanical principles (Rico‐Guevara, Fan, & Rubega, 2015) that necessitate testing in the wild. Hummingbirds may visit a flower at intervals from 10 minutes to a few hours (Araujo & Sazima, 2003; Rodrigues & Rodrigues, 2014), so camera trapping becomes imperative to collect data without researcher‐intensive monitoring. A camera trap that can capture hummingbirds visiting wild flowers requires: (1) detection of small animals, (2) high‐speed video, and (3) tele‐macro functionality (close‐up videos from a distance).

We aimed to quantify a hummingbird's net energy gain during a floral visit, for which we needed to measure wing beat frequency to estimate energy expenditure in addition to energy acquired from the nectar (c.f. Anderson, 1991). A hummingbird may completely deplete a flower in less than a second; capturing this process requires fast triggering. With nectar licking rates up to 20 Hz (Rico‐Guevara, 2014) and wingbeat frequencies usually around 20–50 Hz (Altshuler & Dudley, 2003; Hedrick, Tobalske, Ros, Warrick, & Biewener, 2012), high‐speed video (>200 frames/s) is required to count the number of licks and wing beats during a single visit to a flower, information of primary importance to understand their energetics and consequent decision‐making behaviors in nature.

## 2 MATERIALS AND METHODS

### 2.1 Sensor selection and testing

We choose to use PIR motion sensors over the alternatives listed above because they did not need to be in close proximity to the flower, were cheap and easy to deploy, and successfully detected hummingbirds. We assessed their ability to detect hummingbirds by filming each sensor connected to a light‐emitting diode, at different distances (measured with a laser range finder: Simmons 600) at hummingbird feeders and flowers at the Finca Colibrí Gorriazul, a private field station near Fusagasugá, Colombia. PIR sensors successfully detected over 15 species of hummingbird, varying in size and behavior, at both feeders and flowers. Individuals of one of the smallest species (3.5–4 g), *Chaetocercus mulsant* (Table S3) were reliably detected at distances of 50 cm, and individuals of one of the largest species (6–9 g), *Colibri coruscans* (Table S3) were reliably detected at distances of 100 cm.

### 2.2 Triggering mechanism

**Figure 2:**
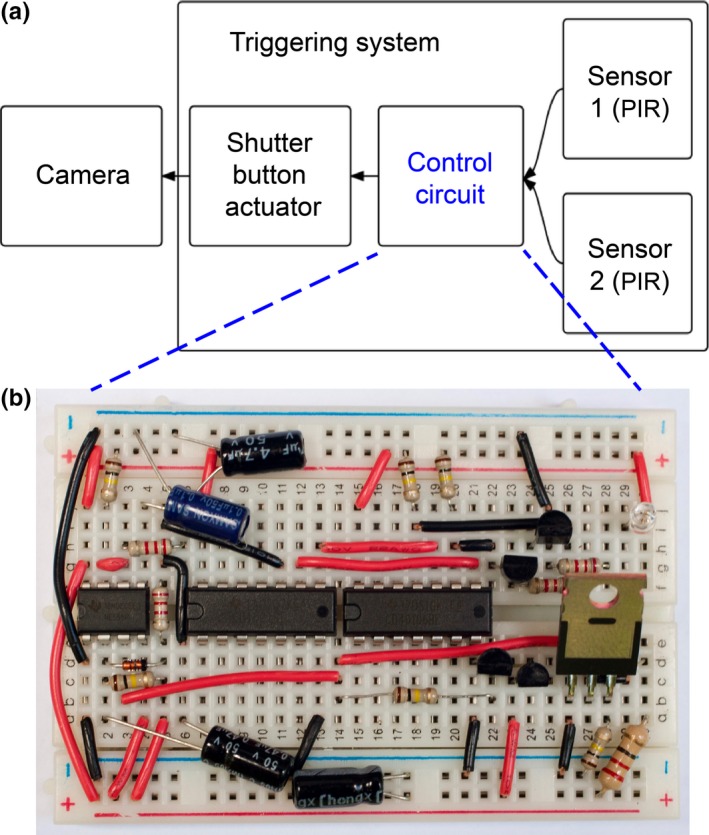
General configuration of the triggering system. (a) Diagram showing, on the right, two passive infrared (PIR) sensors able to detect changes in surface temperature in the scene caused by an animal and to signal the control circuit. Upon receiving a signal from either sensor, the control circuit sends a brief pulse to the shutter button actuator, which mechanically presses the shutter, activating the camera. (b) Close‐up photograph of the control circuit built on a 400 tie‐point breadboard. Breadboards have addresses for rows and columns. A parts list along with their R × C addresses on the breadboard is provided in Table S2 as well as additional photos and diagrams (Figure S1, S2, supplementary circuit diagrams)

**Figure 3:**
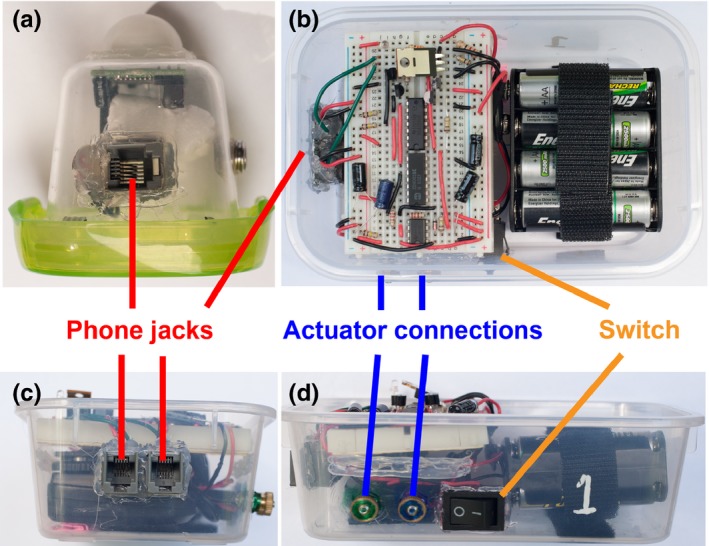
Photos of the sensor and trigger box (three views). (a) Lateral view of a PIR sensor weatherproof module, with the sensor pointing up (white dome) and a phone cord port for connection to the camera trap trigger box labeled in red. On the right side of the sensor, the tripod adapter (1/4‐inch female screw) is visible. (b) Dorsal view of the trigger box with the lid of the weatherproof container removed. On the left is the control circuit (cf. Figure 2b). (c) Lateral view of the trigger box where the weatherproof ports for the sensor phone cord (labeled in red) and the actuator connections (on the right side) are visible. (d) Frontal view of the trigger box in which the power switch (labeled in orange) and the connections for the power wires of the actuator (in blue) can be observed

Our triggering mechanism consisted of two PIR sensors connected to a triggering circuit (Figures 2, 3, S2). When either of the PIR sensors activated, the triggering circuit briefly turned on a mechanical actuator, which manually pushed the shutter button of the camera (Figure 2). The PIR sensors’ retrigger delay was set to 45 s to prevent retriggering before the camera was ready to record again (see supplemental methods); this delay may be unnecessary depending on the study subject and camera selected. Our application also generated a wireless signal to a control box (Fig. S1) some distance away so that a researcher was notified when any traps had triggered to allow for nectar measurements (see below). PIR sensors generally have a range of <7 m for larger‐bodied animals, although with smaller‐bodied animals, such as hummingbirds, their effective range is <1 m. Using multiple external sensors, we were able to optimally position them with respect to the camera, decreasing the likelihood of false negatives. For example, if background vegetation movement was triggering a sensor, we could reposition that sensor (pointing it away from the piece of moving vegetation) without compromising detectability because of the redundancy achieved by having more than one sensor. Furthermore, our standalone sensors (Figure 3) included an option to adjust sensitivity, allowing for fine‐tuning by optimizing the tradeoff between increased sensitivity and false positives. Our final costs per triggering mechanism were <$50 (Table S2) plus the cost of the camera (Table S1, we used pre‐owned cameras of ~$200); more inexpensive than other proposed homemade systems (that generally cost $500–$1,500—Pierce & Pobprasert, 2007; Gula et al., 2010; Steen, 2014) or commercial solutions (that can cost upwards of $1,000–5,000—Meek & Pittet, 2012; Rovero et al., 2013). Triggering mechanisms weighed ~500 g including batteries and the two sensors, much smaller and lighter than many of the alternatives. Additionally, a number of cost‐saving innovations accompany our system and are broadly applicable to any ecological research using homemade electronics or sensors (see supplemental methods).

### 2.3 Camera selection and specifications

More than two frames per wing beat cycle are convenient to estimate hummingbird wing beat frequency (cf. Altshuler & Dudley, 2003). Consequently, we needed cameras with high‐speed video and a prerecord mode (recording a brief amount of video before the shutter is pressed) to compensate for the camera's shutter lag. We compared available high‐speed video cameras to balance affordability of a multicamera setup with the required features, which we include in the Table S1. We opted to use consumer‐grade high‐speed video cameras (cf. Steen, 2014), and after experimenting with different models with our triggering system (first four rows in Table S1, recording length section in Supplement), we chose the Casio EX‐FH20/5, which has already been used for biological research (e.g., Ryerson & Schwenk, 2011). These cameras featured video recording at 210 fps and 480 × 360‐pixel resolution, along with a prerecord mode. We mounted the cameras on light tripods (with triggering system attached) and shielded them with reflective‐layered foam covers, offering rain and sun protection. Cameras were powered externally by two 4xAA battery packs wired in parallel and plugged into the camera's AC adapter port.

### 2.4 System tests

**Figure 4:**
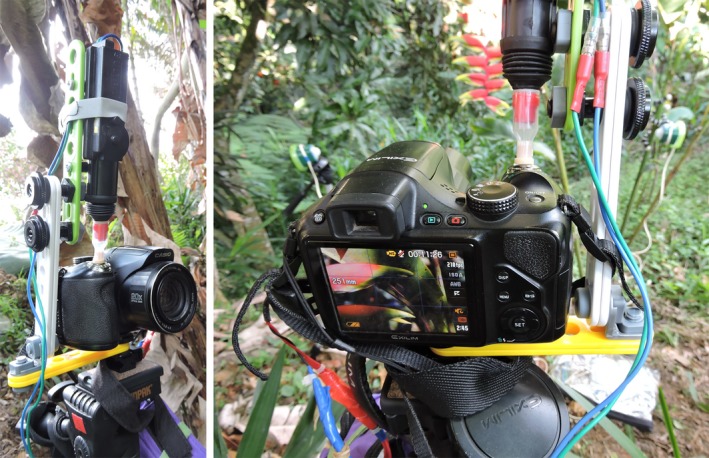
Photographs of the system deployed in the field. The left photograph shows the mounting of the actuator positioned to press the camera's shutter. Our mount used Meccano™ pieces (Meccano S.N., Calais, France), although simple hardware or an articulating arm would suffice. On the right the camera is shown in prerecord mode with two PIR sensors in the background. Camera standby time was extended using an external AA battery pack connected to the camera's power socket (blue and red wires). The trigger box (not visible) is below the camera

We studied hummingbird feeding at Peña del Aserradero Natural Reserve (cloud forest ~2,400 m.a.s.l.) in the Northern Andes of Colombia. We tested the cameras during 3 days (pilot fieldwork), then stopped filming to review the videos and make adjustments (see Section “3”), and finished with seven more days of filming. We deployed camera traps (Figures 4, S3, S4) at focal flowers (May–June 2015), experiencing copious rain, cold (lowest temperatures under 5°C), and intense sun (peak temperatures above 30°C). We collected data simultaneously from four high‐speed cameras situated at focal flowers in different feeding territories. To assess the reliability of the system by documenting missed visits or false positives, backup cameras at each site continuously filmed both the focal flowers and the camera traps at 30 fps (Video S1). We camouflaged all the cameras and systems at the field site (Video S1, Fig. S3), with two researchers alternating waiting for the signals at the base camp and measuring nectar volume and concentrations of the flowers adjacent to the focal ones.

To study energetics, we needed to obtain an estimate of the nectar energy available to a hummingbird at the time of the visit. Therefore, at each camera trap, we bagged a flower next to the focal flower, and immediately after a bird visit, we emulated a “visit” to the bagged flower, and measured nectar volume and concentration. Our triggering system only started recordings at the beginning of a visit; therefore, recordings were stopped manually while researchers measured nectar. We reached each camera location within 1—2 min and did not record hummingbirds re‐visiting the focal flower during these intervals. As triggering systems sent a wireless signal to a base camp when activated, we were able to monitor all four traps simultaneously and immediately emulate flower visits. From the videos, we measured licking rate, bill insertion distance, handling time, amount of nectar collected, nectar properties, and aerodynamic parameters (e.g., wingbeats/s). These allowed us to obtain extraction efficiency (μl/s), energy content in the nectar consumed (cal/μl), and net energy gain (by subtracting the costs of hovering).

## 3 RESULTS

We manually reviewed the pilot fieldwork videos from the backup cameras at 3× speed and compared the number of visits with those captured by our systems. There were 35 hummingbird visits to the focal flowers (e.g., Video S2), and the cameras were triggered 60 times; of these 60, 34 were recordings of actual visits, and 26 were videos of the focal flower without a visiting hummingbird (false positives). One hummingbird visit did not trigger our systems (a false negative) (Table 1). This false negative occurred when a hummingbird arrived during the 45 s trigger delay immediately following a false positive triggered by wind. False positives occurred in only one location and only during the afternoons; studying the backup videos we conjectured that all were caused by strong wind moving vegetation. This location was particularly exposed and windy in the afternoons compared to other locations. Review of backup videos showed that, despite the camouflage, the hummingbirds inspected the cameras and sensors. Nevertheless, after an initial inspection (Video S1), all hummingbirds visited the focal flower and did not inspect them the second and third days. Actuator motion and sound were minimal and occurred away from the flower, provoking no observable behavioral changes in the hummingbirds. Following pilot fieldwork (Figure 4), we performed a series of fixes that minimized the false positives through trial and error at the problematic windy location. We greatly reduced the false positives by repositioning the sensors (away from the piece of vegetation previously triggering them) and decreasing their sensitivity (to ignore background vegetation movement detection), minimizing triggering by vegetation moving the wind. In addition, we enhanced the quality of the data from the videos collected through the triggering systems by improving the zoom and framing (to capture both hummingbird hovering and feeding), and accounted for lighting changes throughout the day (avoiding dark recordings). We also discarded videos from the first day to minimize observer effects.

**Table 1:** Performance of the systems. The number of correctly captured visits, false positives, and false negatives are shown for both our initial pilot fieldwork, and after adjusting locations and PIR sensitivity to minimize false positives. Percentages in parentheses are shown relative to total triggers or total visits, denoting rates of true positives, false positives, and false negatives

	Initial pilot			After adjustment		
	Triggered	Not triggered	Total	Triggered	Not triggered	Total
Visit	34 (57%)	1 (3%)	35	107 (87%)	0 (0%)	107
No visit	26 (43%)	—	26	16 (13%)	—	16
Total	60	1	61	123	0	123

In the extended fieldwork, we documented 107 floral visits by hummingbirds in high‐speed video: There were no false negatives, and we only recorded 16 false positives (Table 1). We collected data on visits to eight plant species by 11 species of hummingbirds (Table S3). The lack of false negatives is a testament to the usefulness of multiple external PIR sensors for capturing hummingbirds, and our final false positive rate of <15% is trivial in comparison to the benefits of our system. It took less than a minute to review one of the false‐positive videos (for a total of about 15 min in the extended fieldwork phase), but about 180 hr to visually review the videos from the continuously filming backup cameras at 3× speed for the same fieldwork phase. By using macro, backlit‐filming techniques (cf. Rico‐Guevara, 2014), we visualized and measured the amount of nectar inside flowers, and tracked the bill and tongue inside the corolla. Through this combination of automated macro, high‐speed, and backlit videography, we were able to observe what was previously unobservable–wild hummingbirds depleting nectar inside flowers.

Our triggering system drew 15 mA of current, with a one‐second 350 mA pulse when triggered. We were able to run each trigger on one set of batteries (8xAA) for the entire study (~100 hr). Battery life depends somewhat on the rate of triggering, but 140–160 hr is reasonable for 2,500 mAh AA batteries and greatly exceeds that of the cameras, even when using external battery packs. The external battery packs for the cameras were changed every 12–15 hr of monitoring and never fully drained. However, their run time was considerably less than that of the triggering mechanisms. Therefore, battery life of the entire system is generally dependent on the chosen camera, not the triggering mechanism.

## 4 DISCUSSION

Filming animal behaviors in their natural environment, while minimizing observer disruption is costly and time‐consuming and camera traps help solve this problem. However, the flexibility of available camera traps is limited, and no options exist for filming high‐speed video of small‐bodied animals such as hummingbirds. We solved this logistical challenge by splitting the camera trap into two parts: the camera and the triggering mechanism. We designed a system that could mechanically trigger specialized cameras and receive input from multiple external sensors positioned separately from the camera, all while being cheap, portable, weatherproof, battery‐efficient, and easy to upgrade. We were able to independently pick the ideal camera and sensor configuration for our application, allowing more control in picking critical camera features such as video frame rate and prerecord mode, and more flexibility with designing sensor configurations to optimize sensitivity. Our system simply presses the camera's shutter button; therefore, cameras can be upgraded and recoupled. The system can also be adapted for cameras with remote triggering by closing the camera's remote trigger switch instead of operating an actuator.

While our application is unique in using high‐speed video camera traps, the main novelty of our design is the decoupling of camera and triggering system, increasing camera trap flexibility. While we used high‐speed video cameras, the system could be adapted to other specialized cameras such as starlight or thermal cameras to study nocturnal animals, including cameras that researchers already own or those with future technological advances. While we used PIR sensors to detect movement, the design is not limited to PIR, and other sensors for light, color, or sound could be employed to trigger the camera instead of or in combination with PIR sensors.

Alternatives to our approach include several technologies already used for studying small animals. Camera traps triggered by AIR sensors (Hernandez et al., 1997) and mini‐DVR video recorders or cameras with video motion detection that start recording when motion in the video is detected (e.g., auto‐record mode on JVC GC‐PX100 or surveillance software such as Scene Analyzer™, i‐PRO SmartHD™, iSpy) are appropriate (Bolton et al., 2007; Kross & Nelson, 2011). So are computer vision algorithms (e.g., Anandan, Bergen, Hanna, & Hingorani, 1993; Joshi & Thakore, 2012; Nordlund & Uhlin, 1996; Zeljkovic, 2013) that have recently been applied to biological studies to filter video for animal activity after recording (e.g., Dell et al., 2014; Weinstein, 2014). However, none of these systems currently support a wide array of cameras, including any with high‐speed video. Most cost in excess of $500 and many use heavy 12 V batteries. We found the auto‐record mode of many video motion detection solutions was too slow to capture the start of a visit by hummingbirds. Computer vision algorithms require continuous prerecorded high‐speed video and acquiring this under field conditions faces significant drawbacks such as short camera battery life and storage problems (a 32 GB memory card lasts 2 hr). Therefore, our solution allows for cost‐effective camera trapping with more functionality than previously possible. One limitation of our system was the high maintenance level of the cameras we used. Cameras needed to be protected with rainproof covers, were turned off at night, and were not deployed for long periods of time. One advantage of commercial camera traps is that they are completely weatherproof and designed for deployments of weeks or months. These features are not useful when filming hummingbirds, due to high turnover rates of inflorescences. Nevertheless, if researchers require longer filming periods, we recommend weatherproof cameras (e.g., GoPro^®^), that can maintain standby mode for long durations.

## 5 CONCLUSION

We are unaware of a recent camera trap application for ecological research in which the triggering system was separated from the camera itself. This approach leads fewer design compromises and has the potential to minimize the limitations of many extant camera traps (Meek & Pittet, 2012; Rovero et al., 2013). In our application, we were able to use a specialized camera with much improved video features from standard camera traps, situate the camera separately from the triggering sensors, modify the sensors, and add wireless capabilities; all at a much lower cost. Our system used simple integrated circuits, although the Arduino^®^ platform could provide future innovations in camera trapping above and beyond those of our system because of its immense flexibility. Arduinos (and similar microcontrollers) have the capability to set video recording duration, trigger flashes, and weigh the input of multiple sensors in complex ways. They can also collect and store ancillary data with sensors for ambient temperature, humidity, and many other aspects of the environment, while being cheap and power efficient.

## CONFLICT OF INTEREST

None declared.

## AUTHOR CONTRIBUTIONS

AG conceived the camera trap idea. JM built the traps and the control box. AG and JM performed initial tests, and AG conducted field tests in Colombia and analyzed videos. AG and JM co‐wrote the manuscript. Both authors contributed to all drafts and gave final approval for publication.
