# Supplementary material for: Bring your own camera to the trap: An inexpensive, versatile, and portable triggering system tested on wild hummingbirds
Source: Ecol Evol. 2017 May 18;7(13):4592–8. doi: 10.1002/ece3.3040 (PMC5496556; doi:10.1002/ece3.3040)
Supplement: Supplementary file 3 [file ECE3-7-4592-s003.docx]

**Supporting information**

**Table S1.** Comparison among some of the high-speed cameras that can be coupled to the triggering system. Frames per second (fps) are compared in columns 3 to 8. Recording time and Pre-Recording function are listed when the information was available.

| **Camera Model** | **Zoom** | **≥600 fps** | **≥400 fps** | **≥300fps** | **240fps** | **210fps** | **120fps** | **Recording time: Limited by (approx.)** | **Pre-Rec**^1^ | **Price (USD)** |
| --- | --- | --- | --- | --- | --- | --- | --- | --- | --- | --- |
| Casio EX-FH100 | 10X |  | 224x168 (420fps) |  | 448x336 |  | 640x480 | SD card size (20 mins) | Yes | $300 (used) |
| Fuji HS10 | 30X |  | 224x168 (480fps) |  | 442x332 |  | 640x480 | Int. memory (30 seconds) |  | $350 (used) |
| Casio EX-ZR1000 | 12X |  | 224x160 (480fps) |  | 512x384 |  | 640x480 | SD card size (20 mins) | Yes | $400 (used) |
| Casio EX-FH20 | 20X |  | 224x160 (420fps) |  |  | 480x360 |  | SD card size (20 mins) | Yes | $400 (used) |
| Canon PowerShot SX50 | 50X |  |  |  | 320x240 |  | 640x480 | SD card size (26 -200 mins) |  | $400 |
| Canon PowerShot SX40 | 35X |  |  |  | 320x240 |  | 640x480 | SD card size (12 -90 mins) |  | $450 |
| JVC GC-PX10 | 10X |  |  | 640x360 |  |  |  | SD card size (2 hours) |  | $900 |
| JVC GC-PX100 | 16X | 320x176 | 320x176 (420fps) | 640x360 (lower res) | 640x360 |  | 640x360 | SD card size (2 hours) |  | $999 |
| Casio EX-F1 | 12X | 432x192 |  | 512x384 |  |  |  | Int. memory (7 mins) | Yes | $950 (used) |
| Sony DSC-RX10 II | 25X | 800x270 (1000 fps) | 1136x384 |  | 1676x566 |  |  | Int. memory (4 seconds) | Yes | $1200 |
| FPS1000 | (depends on lens) | 640x480 (1500 fps) | 1280x720 (550fps) | 1280x1024 |  | 1920x1080 |  | Int. memory (100 seconds) |  | $1445 |
|  |  |  |  |  |  |  |  |  |  |  |
| **Specialized cameras** | **Zoom** | **Fps at full res.** |  | **Full resolution** |  | **Power** |  | **Recording time (s)** | **Pre-Rec**^1^ | **Price (USD)** |
| Edgertronic | depends on lens | 579 |  | 1280x1024 |  | 12 Volt |  | 16.7 | Yes | $5495 |
| MV2-D1280 CameraLink |  | 480 |  | 1280x1024 |  | 12 Volt |  |  | Yes | $6520 |
| Trouble Shooter TS1000 |  | 500 |  | 640x480 |  | Batteries |  | 8.7 | Yes | $7000 |
| MemView |  | 530 |  | 1280x1024 |  |  |  | 1.6 | Yes | $8000 |
| Basler Camera link A504 |  | 500 |  | 1280x1024 |  | 12 Volt |  |  | Yes | $8850 |
| Trouble Shooter TSHRMS |  | 500 |  | 1280x1024 |  | Batteries |  | 2 | Yes | $9900 |
| Phantom Miro eX1 |  | 500 |  | 640x480 |  | Batteries |  | 7 | Yes | $9900 |

^1^ This feature is useful for recording quick behaviors (e.g. floral visits by hummingbirds). For the cameras without this feature the auto power off function must be disabled in order to function properly with the triggering system. All the cameras listed here, that do not have pre-rec function, have the option to disable the auto power down.

**Table S2.** List of required parts and their respective costs for triggering circuits (1). and control box (2). Each triggering circuit cost ~$40, and a large portion of that cost was connected to the wireless remote notification capability, which was unique to our application. The addresses of their positions on the breadboard are also provided.

| **(1). Triggering Circuit & Two PIR Sensors** | | | |  | |
| --- | --- | --- | --- | --- | --- |
| **Description** | **Cost** | **#** | **Total** | | **Breadboard Position** |
| 555 Timer - NE555 | $0.33 | 1 | $0.33 | | E1-F4 |
| Quad NOR Gate - CD4001BE | $0.75 | 1 | $0.75 | | E7-F13 |
| Hex Schmitt Trigger NOT Gate - CD40106UBE | $0.50 | 1 | $0.50 | | E15-F21 |
| PIR sensor - DYP-ME003 | $2.52 | 2 | $5.04 | | (+)-(-)-J27, (+)-(-)-J28 |
| Power MOSFET - IR530 | $0.80 | 1 | $0.80 | | B26-B29 |
| Diodes 1N4148 | $0.03 | 2 | $0.06 | | D2-D5, D27-D30 |
| NPN Transistor 2N4401 | $0.07 | 3 | $0.21 | | H24-H26, G22-G24, D21-D22-D24 |
| PNP Transistor 2N4403 | $0.07 | 1 | $0.07 | | D23-D25 |
| 100k Resistor | $0.02 | 6 | $0.12 | | (+)-F2, (+)-F18, (+)-F20, C2-C5, B14-B22, (-)-A28 |
| 2.2k Resistor | $0.02 | 4 | $0.08 | | H3-H6, E5-F5, F23-F27, G25-G28 |
| 330 Resistor | $0.02 | 1 | $0.02 | | E29-F29 |
| 22 Resistor (1 Watt +) | $0.25 | 1 | $0.25 | | (+)-A30 |
| 0.1 uF Capacitor | $0.10 | 1 | $0.10 | | (-)-J4 |
| 0.47 uF Capacitor | $0.10 | 1 | $0.10 | | A2-A6 |
| 4.7 uF Capacitor | $0.10 | 1 | $0.10 | | (-)-A6 |
| 47 uF Capacitor | $0.10 | 1 | $0.10 | | (+)-(-) |
| Pulse LED (3mm) | $0.10 | 1 | $0.10 | | I29-I30 |
| Power Switch | $0.60 | 1 | $0.60 | | (+)-(+) |
| Project Enclosure (butter container) | $2.44 | 1 | $2.44 | |  |
| Breadboard (400 tie-point) | $4.35 | 1 | $4.35 | |  |
| 9V battery clip | $0.13 | 1 | $0.13 | |  |
| IR Sensor Cables (Phone cables) | $0.42 | 2 | $0.84 | |  |
| Camera Flash Transmitter/Receiver (optional) | $12.99 | 1 | $12.99 | | (+)-A25 |
| Car Power Door Lock Actuator (12v) | $3.15 | 1 | $3.15 | | A25-C30 |
| Phone Jacks | $0.40 | 4 | $1.60 | |  |
| Sensor Housing (butter containers) | $3.00 | 2 | $6.00 | |  |
| Velcro (per ft.) | $1.73 | 1 | $1.73 | |  |
| 8AA Battery Holder with 9v battery clip | $2.44 | 1 | $2.44 | |  |
| Triggering Circuit Total |  |  | $45.00 | |  |
|  | | | |  | |
|  | | | |  | |
| **(2). Control Box (For 4 triggering systems)** | | | |  | |
| **Description** | **Cost** | **#** | **Total** | | **Breadboard Position** |
| Breadboard (400 tie-point) | $4.35 | 1 | $4.35 | |  |
| PNP Transistor 2N4403 | $0.07 | 4 | $0.28 | | D9-D11, H9-H11, D20-D22, H20-H22 |
| NPN Transistor 2N4401 | $0.07 | 4 | $0.28 | | C10-C12-C13, I10-I12-I13, C18-C19-C21, I18-I19-I21 |
| Dual D-Type Flip Flop - CD4013BE | $0.50 | 2 | $1.00 | | E2-F8, F29-E23 |
| Indicator LEDs (5mm Red) | $0.09 | 4 | $0.36 | | (-)-D1, (-)-G1, (-)-D30, (-)-G30 |
| 47 uF Capacitor | $0.10 | 1 | $0.10 | | (+)-(-) |
| Project enclosure (butter container) | $4.03 | 1 | $4.03 | |  |
| Reset Switch | $0.28 | 4 | $1.12 | | (+)-C7, (+)-H8, (+)-C23, (+)-H24 |
| Power Switch | $0.60 | 1 | $0.60 | | (+)-(+) |
| 330 Ohm Resistor | $0.02 | 4 | $0.08 | | B1-D3, H1-H3, C27-C30, G28-I30 |
| 100K Resistor | $0.02 | 12 | $0.24 | | (-)-A5, (-)-A7, (-)-A23,(-)-A25, (-)-J6, (-)-J8, (-)-J24, (-)-J26, C12-E14, H12-F14, E17-C19, F17-H19 |
| Camera Flash Receiver (included w/trigger cost) | $0.00 | 4 | $0.00 | | C13-C14, C17-C18, H13-H14, H17-H18 |
| LED Holder (5mm) | $0.25 | 4 | $1.00 | |  |
| Control Box Total |  |  | $13.44 | |  |

**Table S3.** Hummingbird species (by clade) and plants visited in events recorded by our high-speed camera traps.

| **Clade** | **Species** | **Common name** | **Plants visited** |
| --- | --- | --- | --- |
| Hermits | *Phaethornis guy* | Green hermit | *Heliconia rostrata* |
| Mangoes | *Colibri cyanotus* | Lesser Violetear | *Cavendishia nitida*  *Palicourea angustifolia*  *Palicourea demissa*  *Barnadesia spinosa*  *Fuchsia boliviana*  *Bomarea multiflora* |
|  | *Colibri coruscans* | Sparkling Violetear | *Bomarea multiflora*  *Streptosolen jamesonii* |
| Coquettes | *Heliangelus exortis* | Tourmaline Sunangel | *Barnadesia spinosa*  *Palicourea angustifolia* |
|  | *Metallura tyrianthina* | Tyrian Metaltail | *Palicourea demissa*  *Bomarea multiflora* |
| Brilliants | *Eriocnemis vestita* | Glowing Puffleg | *Cavendishia nitida* |
|  | *Coeligena torquata* | Collared Inca | *Cavendishia nitida*  *Palicourea demissa* |
|  | *Adelomyia melanogenys* | Speckled Hummingbird | *Palicourea demissa*  *Barnadesia spinosa*  *Bomarea multiflora* |
|  | *Boissonneaua flavescens* | Buff-tailed Coronet | *Barnadesia spinosa* |
| Bees | *Chaetocercus mulsanti* | White-bellied Woodstar | *Barnadesia spinosa* |
| Emeralds | *Amazilia cyanifrons* | Indigo-capped Hummingbird | *Fuchsia boliviana*  *Streptosolen jamesonii* |

**
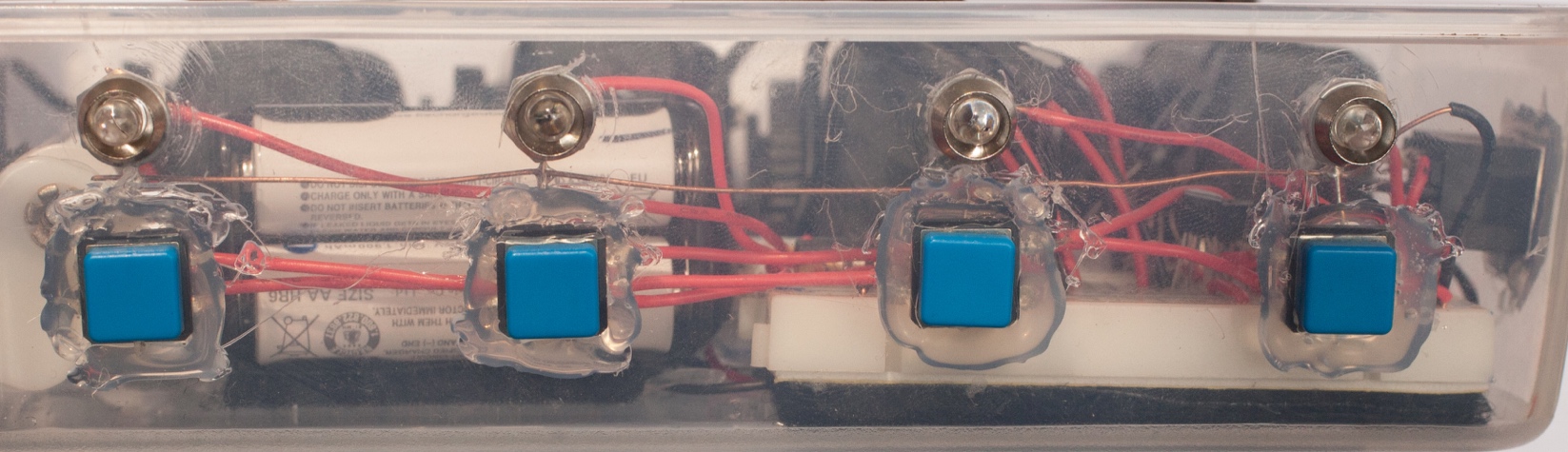
**
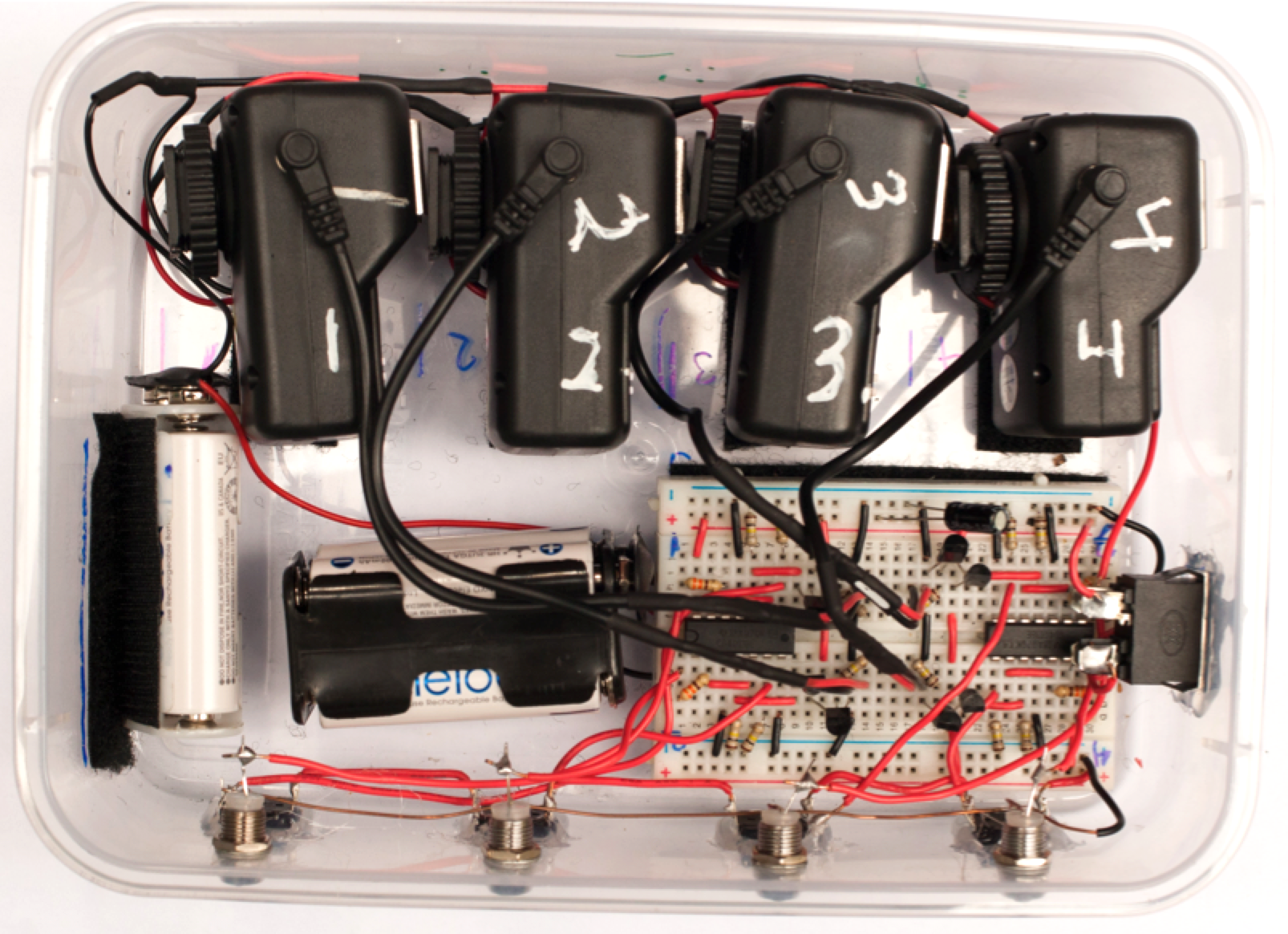


**Figure S1. Photographs of the wireless signal control box.** The top photograph is a dorsal view of the inside of the control box, showing the circuit, batteries, and four camera flash receivers. The bottom photograph shows a side view LEDs mounted above reset switches. When a trap is triggered it sends a signal to its wireless receiver, which turns on the appropriate LED. Pushing the switch below the LED resets it. Higher resolution photos of the circuit are available on [figshare](https://figshare.com/projects/Hummingbird_Camera_Trap/20047) (see below).


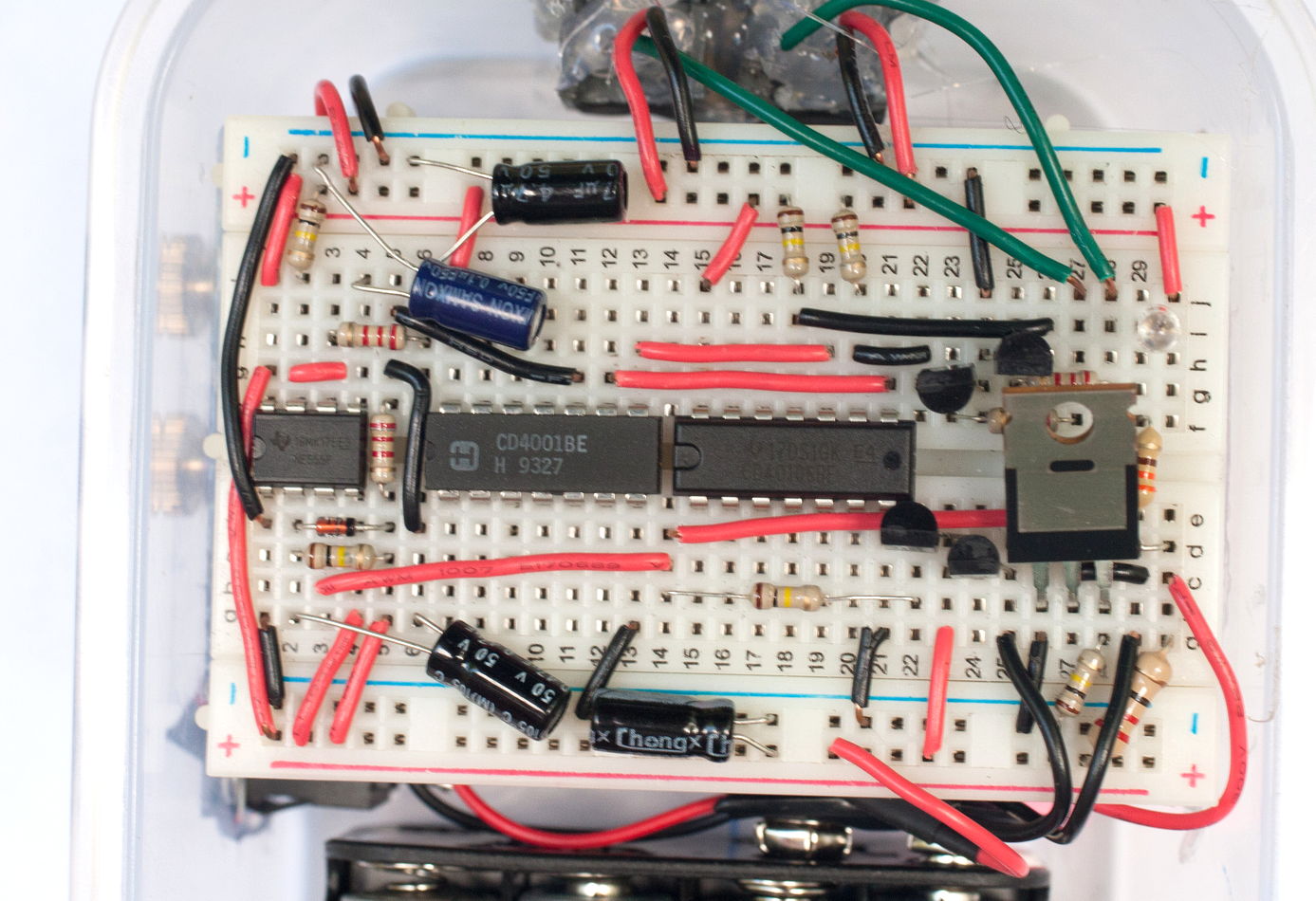


**Figure S2. Close-up of the triggering circuit.** This used a 400-point breadboard, and the circuit can be copied by inserting parts into the same holes. See circuit diagrams and Table S2 for more details. Nearly every component is shown, though the MOSFET obstructs a diode from D27-D30 (cathode), and the red wire from D14-E28. Power from batteries is supplied to the (+) and (-) rails in the top left corner. The two sensors connect at the top, with their signal wires connecting to J27 and J28. The output to the shutter actuator runs through wires connected to A29 and C30. The integrated circuits in the center are from left to right: NE555, CD4001BE, CD40106UBE. The additional wires at the bottom connected to (+) and A25 lead to the wireless camera flash transmitter. By convention, red wires carry (+) and black wires carry (-).

**
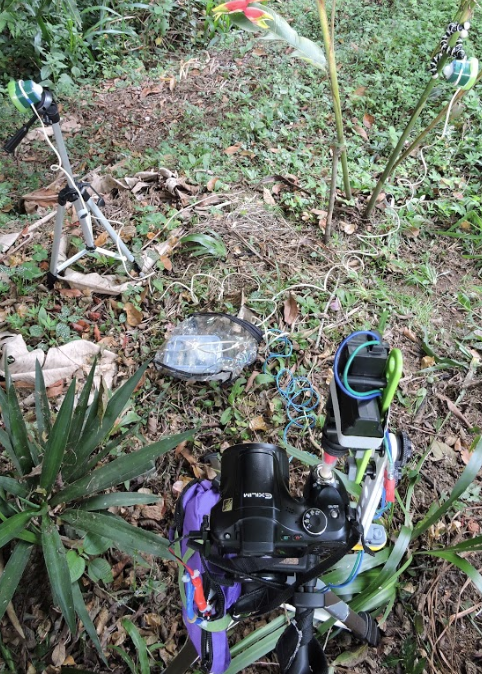

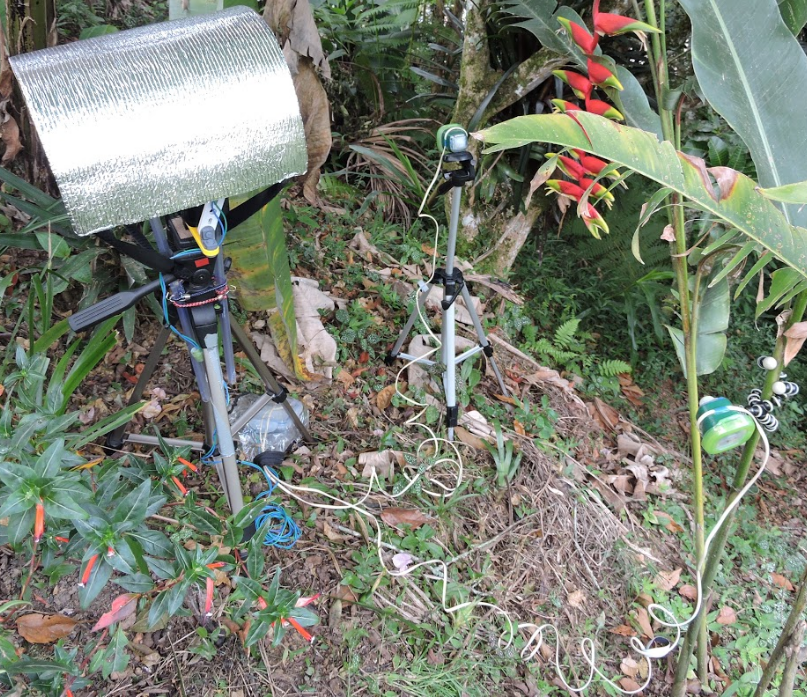
**

**Figure S3. Photos of the triggering system in action.** On the left, the system deployed with the reflective foam cover. One PIR sensor is held by a gorilla pod on vegetation (right), and the other mounted on a light tripod (center, top); the final arrangement varies on each site. On the right, a top view without the cover to note the placement of the actuator pushing the shutter button on top of the camera. The triggering box is additionally protected by a plastic bag. All of this could be covered by a camouflage cloth or pieces of vegetation.


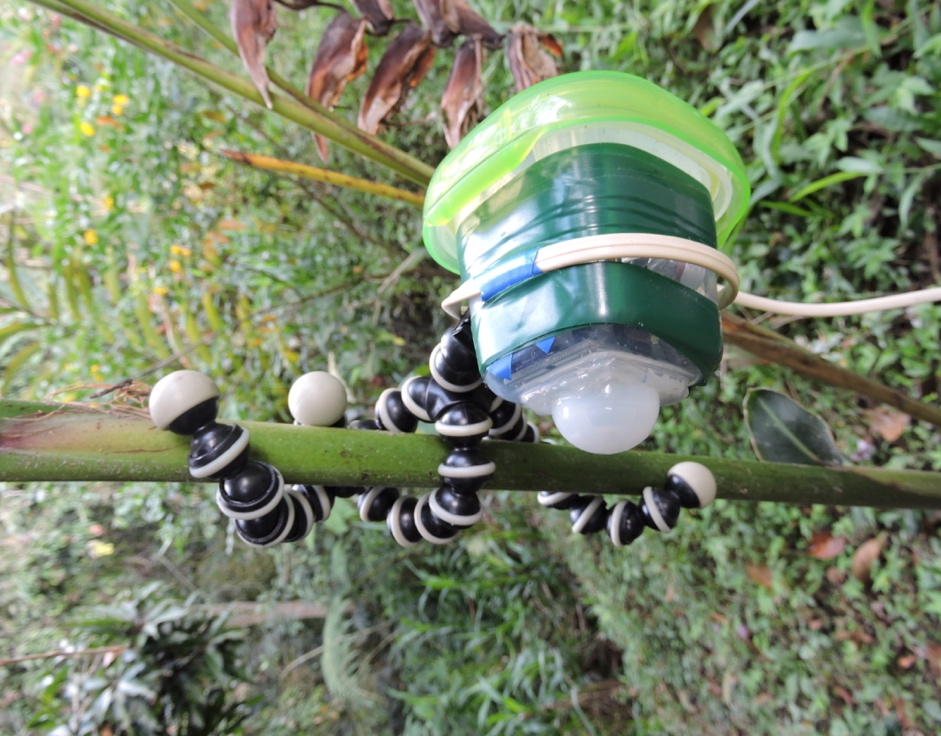

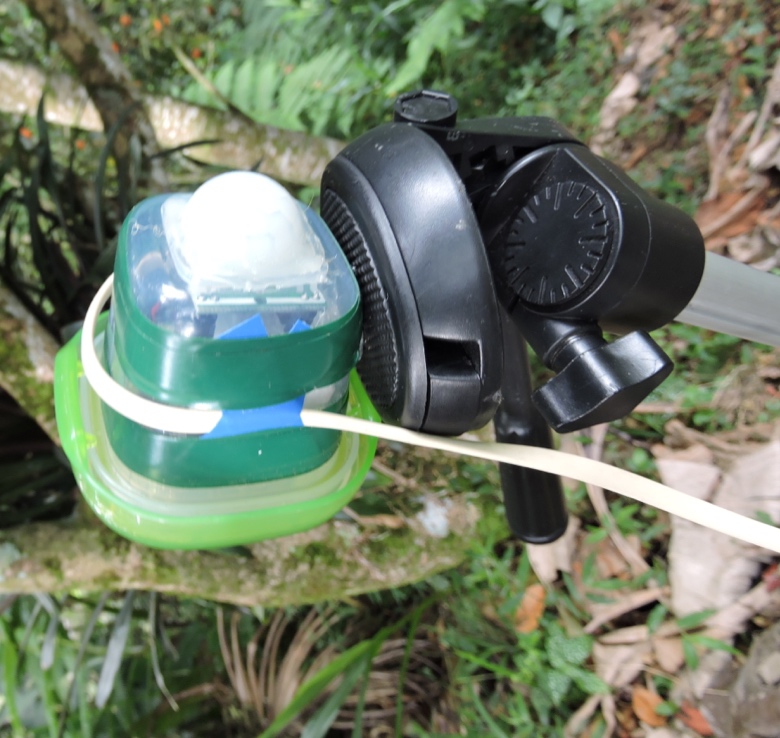

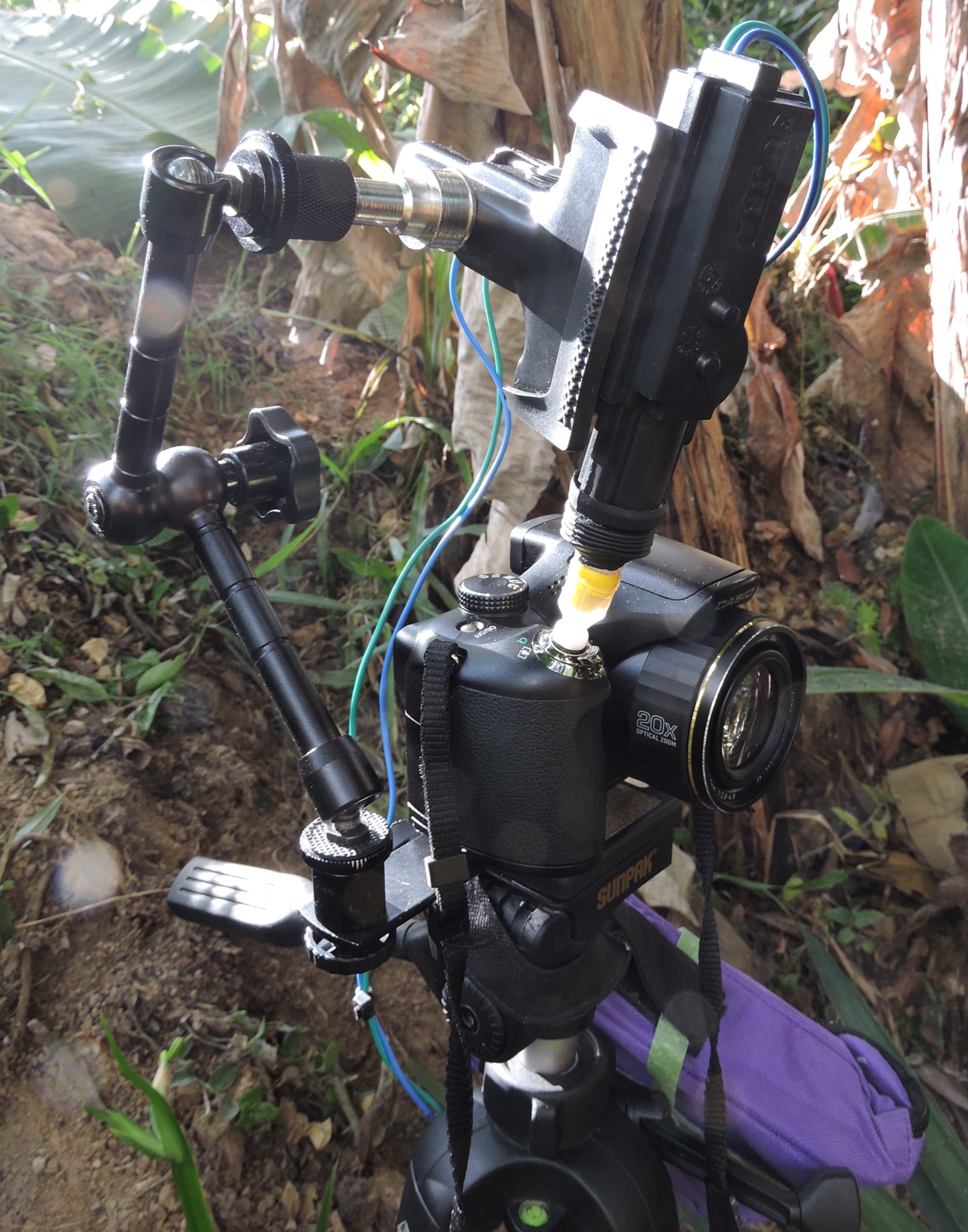


**Figure S4.** **Alternative mountings of actuator and sensors.** On the left, detail of the actuator attachment using easily adjustable, and available off the shelf, articulating arms and clamps (~$15) instead of Meccano™ (http://www.meccano.com) pieces. On the right, two alternative mounts for the PIR sensor units, they are light enough to attach to low cost gorillapods (~$3) that wrap to branches and stems, but they can be attached to any standard 1/4-inch tripod mounting plates. Note that the cable is wrapped around the unit to prevent misconnections by pulling the plug during sensor adjustment.

**
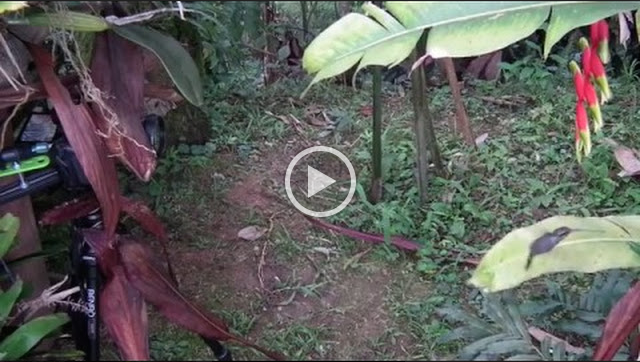
**

**Video S1 (Backup camera video.mov).** Example video of the triggering system in action taken by a backup camera. The hummingbird triggers the trap at 0.01 and the door lock actuator can be seen pushing the camera’s shutter button.

**
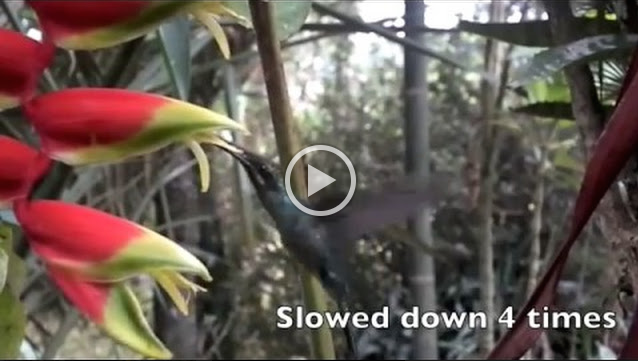
**

**Video S2 (Example camera trap video.mov).** This shows an example video of a Green Hermit (*Phaethornis guy*) visiting a *Heliconia rostrata* flower that we were able to capture using our camera trap. The video has been slowed down to 1/4^th^ and 1/16^th^ speed to facilitate more detailed viewing.

**Further Supplementary Information**

*Cost-saving innovations incorporated in the triggering systems*

1) For enclosures of the control circuit and PIR sensors, we used plastic butter containers with a rubber gasket built into the lid, making them waterproof. Waterproofing can be preserved after cutting holes for switches and inputs by covering the inner parts of these areas with hot glue.

2) We used prototyping ‘breadboards’ rather than fabricating printed circuit boards and soldering components onto them. Breadboards are cheaper, take far less skill to assemble, and allow flexibility for design change or additional features. Wires and components were simply plugged into the board; careful wiring minimizes the chance of wires pulling out of the breadboard sockets. If some degree of permanence is needed, printed circuit boards with the same pattern as the breadboard can be produced.

3) Different components of camera traps require different voltages. While it is trivial to lower voltages for parts of a circuit (by turning some of the electricity into heat), this part of the circuit must run continuously and small batteries such as AA are thus depleted quickly. Thus large and heavy 12v batteries are required for appreciable battery life in the field. We circumvent this by using CMOS 4000 series integrated circuits, which can accommodate a wide range of voltages, usually anywhere from 3-15 volts. We used automotive door lock actuators to trigger the camera (requiring a minimum of about 8 volts); therefore the system can work anywhere from 8-15 volts, allowing for considerable flexibility with battery or power supply configurations without any loss of efficiency through voltage conversion. The CMOS 4000 also has a low standby current draw. Our entire circuit uses only 15-20 milliamps while waiting for a sensor to trigger; when a pulse is sent this briefly jumps to ~350-400 milliamps for half a second.

4) The automotive door lock actuators that push the camera’s shutter button exert more force while using less power and lower voltages than solenoids as they are motor-driven. Ours functioned well with a current draw of only 350-400 milliamps and a minimum of about 8 volts.

5) Cables connecting PIR sensors to the control circuit were made from phone cords, with sockets installed in the sensor and control circuit enclosures. Phone cords, with four internal wires, are less expensive and neater than most wire sources and are easy to find in various lengths.

6) Most radio remote transmitter or transceiver systems cost $20-40 and do not have the ability to run on the separate channels needed to monitor multiple traps simultaneously. Camera flash remote trigger sets (with a transmitter and receiver) cost $10 and support 4-16 separate channels, allowing 4 or more camera traps to operate in the same area. Although their range is somewhat limited, additional sets of transmitter + receiver can be connected together, acting as repeaters. While these are sometimes prone to crosstalk when operating four at a time, they represent a very low-cost solution for sending a simple pulse signal wirelessly.

*Building the triggering system*

Copying our system can be accomplished by building the circuit detailed in the circuit diagrams and Fig. S2. If using a commonly available 400 tie-point breadboard shown in Fig. S2, one can simply plug in components and wires into the same addresses (Columns A-J, Rows 1-30) as in Fig. S2. The addresses of each component are provided in Table S2. If needed, more photos are available at <https://figshare.com/projects/Hummingbird_Camera_Trap/20047>.

First, cut wires to size and install on the breadboard in the same locations as in the photos (Fig 2, Fig. S2, figshare). Color is unimportant, though red and black were used to signify positive and negative voltage. Next, add the other components according to Table S2. Finally, build the enclosure and add the wires external to the breadboard (Fig. 3, Fig. S2).

For some circuit components, the polarity or direction they are plugged into the breadboard is important, and they should be plugged in accordingly. This is true of capacitors, diodes, transistors, ICs, and the MOSFET. In most cases, the polarity can be seen in Fig S2: observing the black band on diodes, the flat side of transistors, and the side with the notch for ICs. In other cases, we refer the reader to the circuit diagrams to get the correct polarity.

The triggering system then also needs a case, a power supply, sensor connections, a connection to the shutter actuator, and a means of mounting and positioning the actuator to be able to push the camera’s shutter. We leave these to the ingenuity of the reader, though our system can be used as a template (e.g. Fig 3, Fig 4, Table S2, Fig S2, Fig S3, Fig. S4).

*Battery performance*

We used the same set of eight rechargeable AA batteries for the pilot fieldwork and data collection, testing for proper functioning of the triggering system every day before deploying it in the field. Since we never changed the batteries and the systems continued to work, we estimate that under normal use, a set of batteries would last between one and two weeks, with the majority of the current drain being standby current.

*Ending recordings & recording length*

Our triggering systems were originally designed for use with the Fuji HS10 camera (Table S1), which had better frame rate and zoom than the Casio EX-FH10. The Fuji camera only needed the shutter to be pressed at the start of recording; recordings were limited to 30 seconds. We allotted 15 extra seconds for the camera to transfer the recording from the internal memory to the SD card and be ready for the next recording, for a total of 45 seconds of programmed delay between triggers in our system. However, during our sensors and camera models trial phase (prior to field testing) we found that the pre-record option offered by the Casio EX-FH20 camera improved the capture of some hummingbirds in which we could not predict the path they would use to approach the flower; therefore, the Casio cameras were used instead. The Casio cameras did not stop recording after 30 seconds and since our triggering system only sent a single pulse at the start of a visit, recording was stopped manually (after 1-2 mins) when a researcher visited the flower adjacent to the focal flower to measure nectar properties (see methods). For situations where this is inconvenient or infeasible, the best option is probably to use an Arduino or other microcontroller to generate two pulses, one at the beginning of a visit and one at the end, after a desired amount of time (all visits in our study were completed after only a few seconds). The microcontroller would connect directly to the PIR sensors and the MOSFET, replacing the NE555, CD4001, and CD40106 chips.

*Wireless notification system*

The wireless notification system using camera flash remotes worked well, though we observed some cases where triggering one camera trap sent a positive signal for more than one trap (crosstalk). In practice, we found their range to be 20-30 meters, beyond that they required repeaters, which were not always reliable. However, during the testing phase the wireless system box was able to accurately notify us when one of the four deployed triggering systems was activated even within the dense vegetation of the cloud forest.

*Description of control circuit*

What the camera trap circuit does in abstract terms is turn a low voltage signal from a passive infrared (PIR) sensor into a short pulse of much higher voltage and amperage, sufficient to operate an automotive door lock actuator and thus press the camera’s shutter button. When one of these PIR sensors is triggered, it sends a 3.3v positive current through it’s OUT pin. This is then brought up to the operating voltage, amplified and inverted to negative current by an NPN transistor (2N4401). The signal then flows to a Hex Schmitt Inverter (CD40106) integrated circuit, which inverts it back to positive current and levels out the signal by requiring a threshold voltage to activate. The modified PIR signals are then sent to a quad 2-input NOR gate (CD4001), which provides a negative trigger current to the 555 timer pulse generator if any sensors were triggered. While we used only two sensors the quad NOR gate can support up to three and a dual 4-input NOR gate such as the CD4002 could support four sensors.

Next, the output of the NOR gate goes to a 555-timer pulse generator circuit. When the NOR gate is outputting positive current, nothing happens, but if a sensor triggers and the NOR gate outputs a negative current, this triggers the pulse generator and sends a positive pulse of about half a second. This pulse turns on the IR530 power MOSFET, which acts as a switch and is able to handle the heavy current load needed to trigger the door lock actuator. The pulse also triggers a camera flash transmitter, sending a remote signal to a control box some distance away.

*Where to buy parts*

Most electronics and mounting components that we used were sourced from eBay. While RadioShack and various online parts suppliers such as mouser.com and digikey.com carry comparable components, they are usually more expensive and often cannot be bought in small quantities.
