# Supplementary figures and images for: Bring your own camera to the trap: An inexpensive, versatile, and portable triggering system tested on wild hummingbirds
Source: Ecol Evol. 2017 May 18;7(13):4592–8. doi: 10.1002/ece3.3040 (PMC5496556; doi:10.1002/ece3.3040)

[illegible]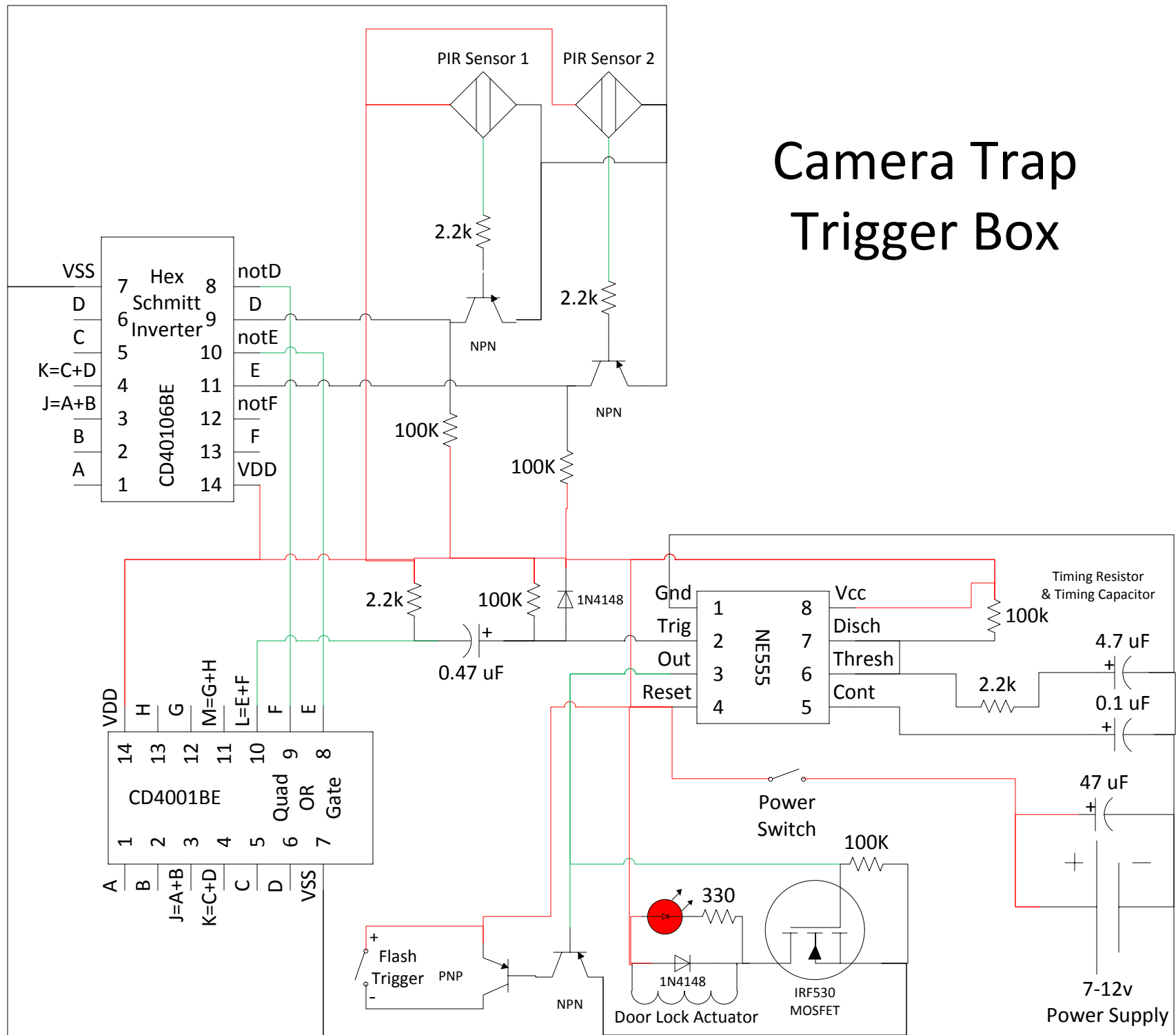

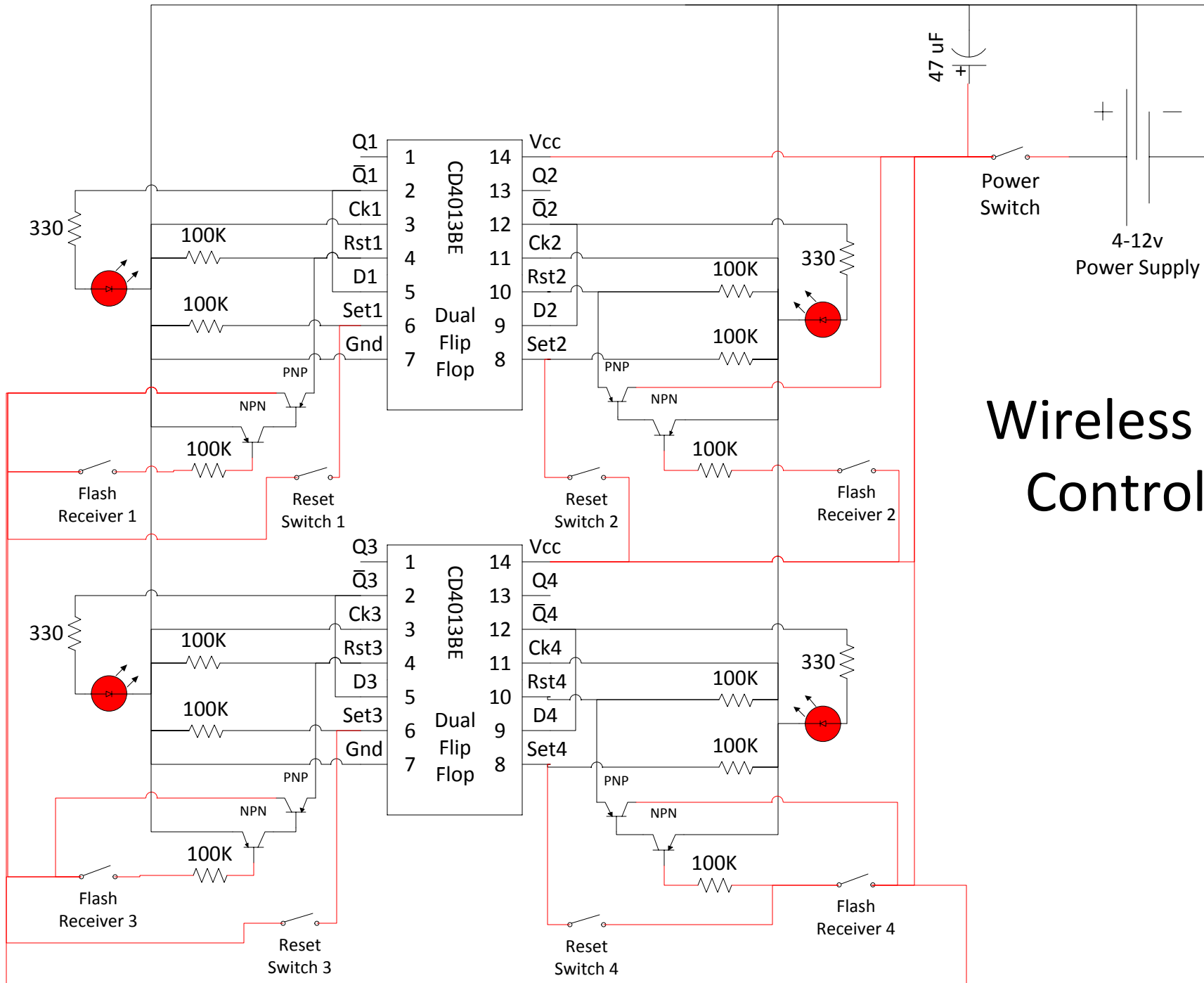

# Wireless Signal Control Box

Supplement: Supplementary file 4 [file ECE3-7-4592-s004.pdf]
